# The recent trends in discrimination and health among ethnic minority adolescents: an integrative review

**DOI:** 10.1186/s12889-025-21729-0

**Published:** 2025-03-04

**Authors:** Sookyung Kim, Hyeonkyeong Lee, Kennedy Diema Konlan

**Affiliations:** 1 School of Nursing, Soonchunhyang University, Cheonan, Republic of Korea; 2 Mo-Im Kim Nursing Research Institute, College of Nursing, Yonsei University, 50 Yonsei-ro, Seodaemun-gu, Seoul, 03722 Republic of Korea; 3 Department of Public Health Nursing, School of Nursing and Midwifery, University of Health and Allied Sciences, Ho, Ghana

**Keywords:** Health, Discrimination, Ethnic minorities, Adolescents, Review

## Abstract

**Background:**

Experiences of racial discrimination during adolescence can negatively affect lifelong health. Although many ethnic minority adolescents face discrimination in common worldwide, there are few updated review studies that explored how discrimination affected health status and behavior among ethnic minority adolescents.

**Methods:**

Comprehensive searches of the PubMed, Embase, PsycINFO, and CINAHL were conducted, integrating keywords about adolescent, ethnic groups, discrimination, and health. The search encompassed articles published between January 2016 and March 2021, following Whittemore and Knafl’s integrative literature review method. Quality appraisal was evaluated by the Risk of Bias Assessment Tool.

**Results:**

After conducting the initial screening of 167 studies, eleven studies met the inclusion criteria and were included in the review. More than 80% of the studies were conducted African-American/Black adolescents in the United States. There were clear associations between group discrimination and mental health problems. Discrimination was also linked to the low overall health, high substance use, high emotional eating, and high behavioral problems, and low ethnic identity. Of the six studies investigating gender differences, two each reported that discrimination was linked to higher substance use in boys and stronger internalizing symptoms in girls.

**Conclusion:**

This integrative review provides insights into the discrimination experiences of ethnic minority adolescents, with particular implications for mental health, overall health, substance use, and behavioral problems. This review contributes evidence for need of integrative health promotion programs to mitigate racial discrimination against ethnic minority adolescents for health equity.

**Supplementary Information:**

The online version contains supplementary material available at 10.1186/s12889-025-21729-0.

## Background

Discrimination is defined as the prejudicial or unfair treatment of people and groups based on factors such as ethnic affiliation or phenotypic characteristics [1], which may be motivated by racism that contains negative emotional actions to racial groups [2]. Ethnic minority groups have been excluded and discriminated against by racial and cultural differences because they are outnumbered and foreign within an extensive range of groups or countries [3]. Evidence has shown that discrimination can cause adverse health impacts in a variety of cultures and countries [1, 4], thereby undermining health equity [5].

Although non-discrimination is being emphasized as the principle of the 2030 Agenda for Sustainable Development Goals [6], adolescents who still experience discrimination are common around the world. In the United States, nearly one-third of adolescents experience discrimination [7]. In Australia, over 30% of adolescents reported on experiences of discrimination including skin colour, language accent, and cultural background [8]. This may be particularly problematic during the critical period of adolescence, in which individuals gain personal independence, forge social relationships, and learn behaviors that will endure the course of life [9]. Moreover, ethnic minority adolescents are at a particularly high risk for health-related challenges, including those pertaining to social exclusion, rejection by [9] community members, and discrimination [9]. This is a serious issue globally, as experiences of discrimination during adolescence can negatively affect health throughout the life cycle [10].

Acknowledging the crucial role of discrimination in the health of ethnic minority groups, professional organizations and institutions have been implementing strategies to improve health care and practice. For example, the National Institutes of Health (NIH) in the United States has formed the UNITE initiative to eradicate racism within biomedical research enterprises [11]. The International Council of Nurses (ICN) expects nursing professionals to be aware of health vulnerabilities that are related to physical, psychosocial, spiritual issues experienced by ethnic minority groups; as such, working to improve the delivery of health care by increasing their cultural competencies and incorporating those elements into practice [12].

According to previous research, a literature review of studies published through the end of 2016 showed that discrimination targeted at adolescents was related to well-being in terms of socioemotional, behavioral, and academic issues [13]. Further, a review of longitudinal studies conducted between 2003 and 2017 showed that adolescents who experienced discrimination during adolescence tended to have a variety of behavioral problems, including increased risk-taking behaviors, substance use, and poor mental health outcomes [14]. In 2022, the World Health Organization emphasized that ethnicminorities’ experiences of discrimination increase their vulnerability to health risk factors, leading to poor health outcomes [15]. This prompted the need to update our knowledge with a review of research conducted in the last five years since 2016. Particularly, while gender differences are a significant in discrimination and health behavior/outcomes [16, 17], and a review [13] revealed significant differences in the race-by-gender moderate effect in the academic aspect on discrimination and well-being among adolescents. Moreover, a systematic review [14] did not address gender differences, leading to limited conclusions concerning the relationship between adolescents’ experiences of discrimination and health according to gender. Therefore, this study needs to explore gender differences in health based on discrimination experiences.

Previous review studies have focused on health issues and discrimination in ethnic minority groups from the general social and epidemiological perspectives, which mainly searched social science databases such as PsycINFO, ERIC [13, 14]. As such, these reviews utilized only a subset of the major sources recommended as biomedical databases, including PubMed (MEDLINE), Embase, CINAHL, PsycINFO, and the U.S. National Library of Medicine’s Cochrane Library, and are therefore limited to fully encompass the necessary evidence. There is insufficient evidence on the effect of discrimination on health including the biomedical aspects, although national institutions such as NIH have urged actions to end racism through a commitment to diverse biomedical professionals including researchers [11]. In addition, the global COVID-19 pandemic resulted in increased racial discrimination [18, 19], and racial bullying of adolescents in schools [20], raising further concern for adolescents discrimination. Since the experience of discrimination among ethnic minority adolescents in adolescence, which is a crucial period of physical and mental health in their entire life, is still high, we require an updated literature review on the discrimination and health of ethnic minority adolescents.

### Aims

This study conducted an integrative review of research from the past five years on adolescent discrimination, its impact on health status and health behaviors, and the gender differences in discrimination on the health of ethnic minority adolescents.

## Methods

Following the integrative review method proposed by Whittemore and Knafl [21], this study searched for and evaluated articles according to five steps: (1) problem identification, (2) literature search, (3) data evaluation, (4) data analysis, and (5) presentation.

### Problem identification

The research question was “How do experiences of discrimination influence health and health behaviors in ethnic minority adolescents?” To address this, the specific approaches were employed: (a) explore relevant study characteristics, (b) identify the influences of discrimination on health status and health behaviors, and (c) explore any gender differences in these health and health behaviors. Health status encompasses both physical and mental well-being including conditions such as depression and anxiety [22]. Health behaviors were defined as behavior patterns and habits related to health maintenance, health improvement, and healeth restoration such as smoking, alcohol use, physical activity [23, 24].

### Literature search

**Fig. 1:**
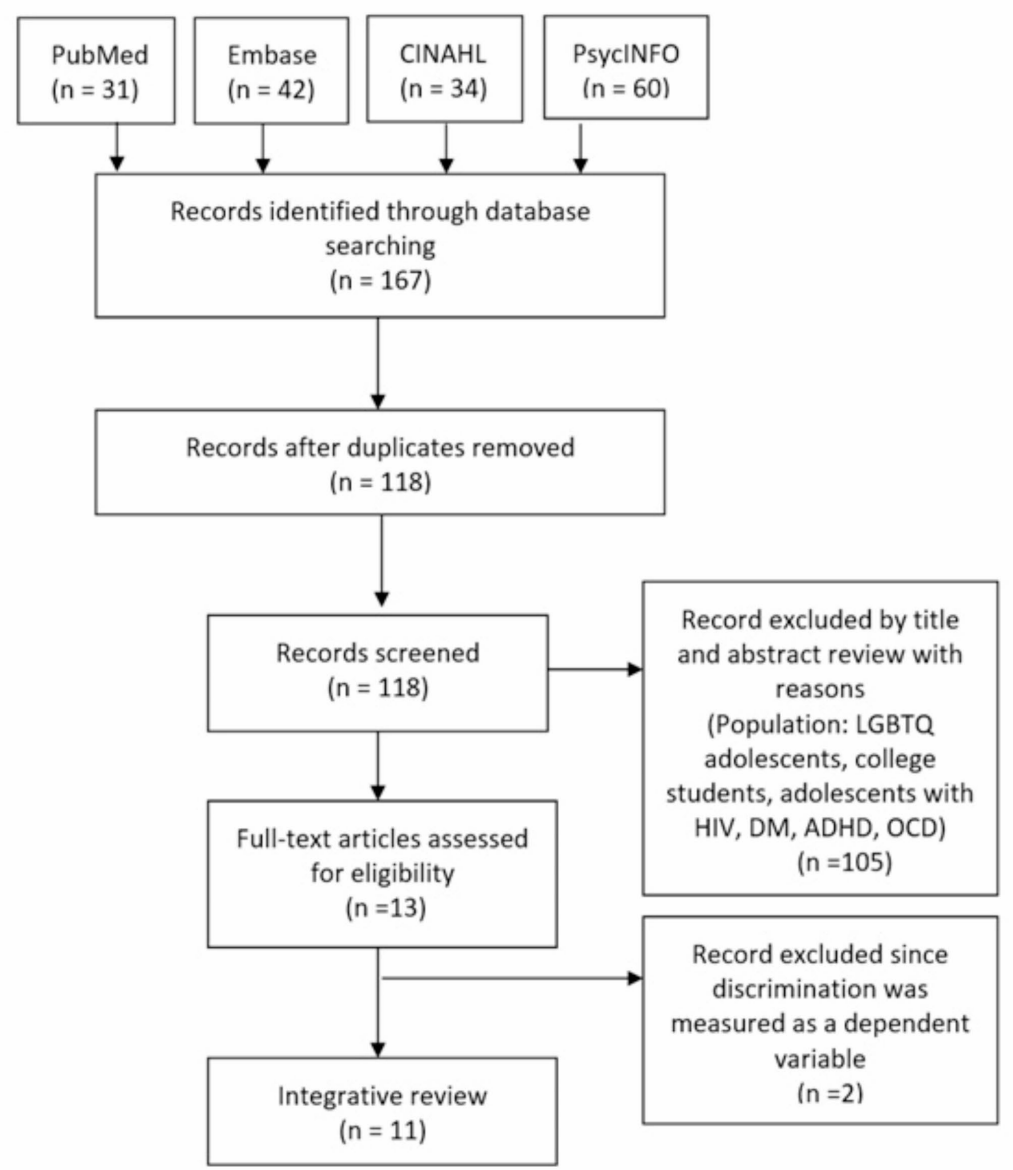
PRISMA flow diagram

We conducted literature searches in four databases (PubMed, Embase, PsycINFO, and CINAHL) using the following keywords: 1) “adolescent,” “ethnic groups,” “discrimination,” and “health”. The search query in PubMed was as follows: ((teen* OR adolescen* OR child* OR youth* OR student*) AND (ethnic groups OR minority groups OR multicultural)) AND ((discrimination OR discrimination, psychological OR social discrimination) AND (health OR health behavior)). Studies were limited to those published between January 2016 and March 2021 and search scope were limited by title/abstract. The keywords were adapted for each database. The search, screening and reporting were guided by the Preferred Reporting Items for Systematic Reviews And Meta- Analysis (PRISMA) framework [25]. The inclusion criteria were as follows: (1) peer-reviewed articles, (2) written in the English language, (3) cross-sectional, retrospective, prospective designs, and (4) topics related to discrimination and health/health behaviors in ethnic minority adolescents. This initially resulted in a total of 167 articles. After removing duplicate articles using EndNote X9 and a manual search, we were left with 118 articles. After further reviewing the titles and abstracts, we excluded 105 articles due to non-applicable populations, including specific groups such as LGBTQ adolescents, adolescents with diseases (e.g., Human Immunodeficiency Virus, Diabetes Mellitus, Attention Deficit Hyperactivity Disorder, Obsessive Compulsive Disorder), and college students. Finally, after reviewing the full texts of the 13 remaining articles, we selected 11 for the analysis. Fig. 1 demonstrates the detailed flow diagram of the literature selection.

### Data evaluation

We investigated the quality of the returned literature via the Risk of Bias Assessment Tool for Non-Randomized Studies (RoBANS) version 2.0. In this context, there were six areas of potential risk, including the selection of participants, confounding variables, measurements of exposure, blindness to outcome assessments, incomplete data, and the reporting of selective results [26]. RoBANS returns three categories of risk ratings, including low risk, high risk, and unclear. In this study, two researchers independently conducted quality appraisals, then reconciled their results to reach a consensus.

### Data analysis

We used a framework for data extraction in which two researchers extracted data from the selected studies based on the following categories: author, year, country, population, age, sample size, study design, data source or sampling, survey respondent, measurements type of discrimination, outcomes and key findings. The extracted data from the two authors were compared, and where discrepancies existed, the third author was consulted. Differences were resolved through consensus.

## Results

### Study characteristics

Of the 11 selected studies, the publication year distribution was as follows: eight studies from 2018 to 2019, two studies from 2020 to 2021 (ending in March), and one study from 2016 to 2017. The majority of studies were conducted in the United States (81.8%), followed by the Netherlands (18.2%). Further, the majority of studies were conducted among African-American/Black adolescents (81.8%), followed by Latino/Hispanic adolescents (45.5%), and biracial/multiracial adolescents (27.3%). As for the research designs, eight studies were cross-sectional (72.7%) and three were longitudinal (27.3%). While the study populations varied in age (range of 6–19 years), most were conducted among individuals aged 13–17 years. Looking at the data types, six studies investigated primary data (54.5%), with sample sizes ranging from 118 to 612 individuals, while fives studies investigated secondary data (45.5%) (e.g., National Survey of Children’s Health), with sample sizes ranging from 1,170 to 95,677 individuals (Table 1). Four studies (36.4%) were sampled from several schools (range of 5-159), one study (9.1%) recruited adolescents who were on probation, and the other study (9.1%) used referral sampling. Two studies (18.2%) using National Survey of Children’s Health data had parent or guardian responses, while the remaining nine studies (81.8%) had adolescent self-report or interview responses (Table 2).

**Table 1:** Characteristics of studies on discrimination and health among ethnic minority adolescents

Category	Description	*n*	%
Published years	2016–2017	1	9.1
	2018–2019	8	72.7
	2020–2021	2	18.2
Country	United States	9	81.8
	Netherlands	2	18.2
Ethnic background^†^	African-American/ Black	9	81.8
	Latino/Hispanic	5	45.5
	White	3	27.3
	Moroccan-Dutch	2	18.2
	Dutch	1	9.1
	Turkish-Dutch	1	9.1
	Surinamese-Dutch	1	9.1
	Antillean-Dutch	1	9.1
	Asian	1	9.1
	Biracial/multiracial	3	27.3
Research design	Cross-sectional study	8	72.7
	Longitudinal study	3	27.3
Data types	Primary data	6	54.5
	Secondary data	5	45.5

^†^Categories are not mutually exclusive

**Table 2:** Summary of studies regarding discrimination and health among ethnic minority adolescents

Author/ Year	Country	Population (Age) Sample	Study design	Data source or Sampling	Survey respondent	Measurements type of discrimiation	Outcomes		Key findings
Adriaanse et al. 2016	Netherlands	Moroccan-Dutch adolescents (9–16 years) *N* = 152	Cross-sectional study	Sampled in eight primary schools and ten secondary schools throughout the Netherlands	Adolescent (self-report)	·Discrimination Questionnaire ·Personal discrimination (skin color, origin, religion) ·Group discrimination (four situations; street, school, shops or by the police)		Psychiatric symptoms, psychiatric disorders	·Psychiatric symptoms (OR^†^ = 2.69, 95% CI^‡^ = 1.13–6.41) and psychiatric disorders (OR = 4.65, 95% CI = 1.37–15.79) were associated with more perceived personal discrimination. ·Psychiatric symptoms (OR = 2.14, 95% CI = 1.35–3.40) and psychiatric disorders (OR = 2.39, 95% CI = 1.27–4.47) were associated with more perceived group discrimination. ·The more perceived group discrimination was associated with more psychiatric symptoms (OR = 2.82, 95% CI = 1.57–5.06).
Assari et al. 2018	United States	Caribbean Black adolescents (13–17 years) *N* = 360	Cross-sectional study	National Survey of American Life-Adolescent Supplement (NSAL-A), 2001–2003	Adolescent (interviewing)	·A modified version of the Everyday Discrimination Scale (e.g., receiving poorer service than other people at restaurants) ·EDS + perceived discrimination by teacher.		Smoking, substance use	· Positive association was found between perceived discrimination and substance use (OR = 1.15, CI = 1.02–1.29). · A significant interaction was found between gender and perceived discrimination on smoking (OR = 1.23, 95% CI = 1.07–1.41) suggesting that the association between perceived discrimination and smoking is larger for male than female Caribbean Black adolescents. · The high levels of discrimination were associated with higher substance use in male Caribbean Black adolescents (OR, 1.32; 95% CI: [1.05, 1.67]).
Colen et al. 2018	United States	African American and Hispanic adolescents (14–21 years) *N* = 9660	Prospective cohort study	1979 National Longitudinal Study of Youth (NLSY)	Adolescent (self-report)	·Experiences of acute discrimination: Major Experiences of Discrimination Scale · Chronic discrimination: Everyday Discrimination Scale		Overall health	· Upwardly mobile African Americans were significantly more likely to experience acute discrimination (B = 0.67, *p* < 0.001), while upwardly mobile Hispanics experienced higher rates of chronic discrimination compared to their socioeconomically stable counterparts (B = -0.86, *p* < 0.05). · After adjusting for acute discrimination, the regression coefficient for Blacks drops by 58% (from − 0.19 to -0.08, *p* < 0.05), losing statistical significance, while the coefficient for Hispanics decreases marginally by 6% (from − 0.34 to -0.32, *p* < 0.05) and remains significant.
Pachter et al. 2018	United States	African-American and Afro- Caribbean adolescents (13–17 years) *N* = 1170	Cross-sectional study	National Survey of American Life-Adolescent Supplement (NSAL-A), 2001–2004	Adolescent (interviewing)	·Everyday Discrimination Scale ·EDS + perceived discrimination by teacher.		Mental health (anxiety, major depressive disorder)	· Ninety point 2% of the African-American and 86.9% Afro-Caribbean adolescent’s samples encountered at least one of the experiences on the Discrimination Scale. · There was no significant difference in perceptions of discrimination by ethnicity. · Discrimination was significantly associated with 12-month major depressive disorder (OR 1.21, 95% CI 1.06–1.36) and 12-month anxiety (OR 1.15, 95% CI 1.00–1.33).
Bouhaddani et al. 2019	Netherlands	Dutch, Moroccan–Dutch, Turkish–Dutch, Surinamese–Dutch, and Antillean–Dutch (13 secondary schools) *N* = 1194	Longitudinal study	Part of the MasterMind project, a school-based screening study of adolescents’ mental health in the Netherlands	Adolescent (self-report)	·Discrimination Questionnaire ·Personal discrimination (skin color, origin, religion) ·Group discrimination (four situations; street, school, shops or by the police)		Prevalence of psychotic experiences	·Perceived personal discrimination was associated with the presence of psychotic experiences including delusional and hallucinatory experiences (OR 2.30, 95% CI 1.22–4.34). ·No significant associations were found between perceived group discrimination and psychotic experiences. ·A weak ethnic identity was associated with higher risk for reporting psychotic experiences (OR 2.04, 95% CI 1.14–3.66), particularly hallucinatory experiences (OR 3.15, 95% CI 1.54–6.44). ·Those feeling marginalized were three times more likely to report psychotic and hallucinatory experiences compared to those with a separated identity (OR 3.17, 95% CI 1.04–9.63) Those with an assimilated identity were three times more likely to report hallucinatory experiences than separated adolescents (OR 3.25, 95% CI 1.30–8.13)
Zapolski et al. 2018	United States	African American (M) in grade = 4 to 12 *N* = 612	Annual cross-sectional study (first of the 5-year series)	Sampled from 159 schools (21 school districts) in a large Midwestern county	Adolescent (self-report)	·A single items of racial discrimination (e.g., In the last year, how often did a kid at my school tease me about my race/ethnicity or the color of my skin? )		Depressive symptoms, anxiety symptoms, substance use	· Separate hierarchical regression analyses revealed that, after controlling for the effect of sex and grade, racial discrimination had a significant effect on depressive symptoms (β = 0.22, *p* < 0.001), on anxiety symptoms (β = 0.20, *p* < 0.001), on substance use (β = 0.15, *p* < 0.001).
Coleman et al. 2019	United States	African American men who were paired with one of their adolescents sons *N* = 118 (father 59, son 59)	Cross sectional study	Referral sampling, Initial recruitment with African American father–son dyads started in Hartford and Windham, CT.	Both father and son	·Everyday Discrimination Scale (e.g., You receive poorer service than other people at restaurants or stores)		Emotional eating, body Mass Index	· Everyday discrimination scores did not differ between fathers and sons. · Only sons had a significant actor effect from discrimination to obesity (β = 0.42, *p* = 0.02). ·African American fathers (β = 1.13, *p* = 0.02) and sons (β = 1.13, *p* = 0.03) that reported more experiences of everyday discrimination reported more emotional eating.
Loyd et al. 2019	United States	Arrested adolescents on probation − 86% African American ‘ (13–18 years) *N* = 173	Cross sectional study	Subset of a federally-funded study testing the efficacy of an HIV prevention program for youth on probation	Adolescent (self-report)	·Interpersonal Ethnic/racial discrimination (ERD): Adolescent Discrimination Distress Index (e.g., hassled by a store clerk or store guard) · Group ERD: scale of ethninc experience (e.g., My ethnic group is often criticized in this country.)		Internalizing symptoms, externalizing behaviors, traumatic stress, emotional dysregulation	· Interpersonal ethnic/racial discrimination was significantly associated with higher internalizing symptoms (B = 7.09, 95% CI = 3.95–10.2, *p* < 0.001) and externalizing behaviors (B = 7.87, 95% CI = 4.39–11.3, *p* < 0.001). The association between interpersonal ethnic/racial discrimination and internalizing symptoms was stronger for girls than boys (B = − 5.12, 95% CI=–9.88 – − 0.35, *p* = 0.035). · There was a significant indirect relation between interpersonal ethnic/ racial discrimination and internalizing symptoms via symptoms of traumatic stress for girls (β = 0.33, 95% CI = 0.16–0.56) and boys (β = 0.19, 95%=CI 0.00–0.36).
Weeks & Sullivan, 2019	United States	Children and youth (6–17 years) *N* = 60,700	Cross sectional study	2011–2012 National Survey of Children’s Health (NSCH)	Parents or guardians	·A single item of racial/ ethnic discrimination (e.g., Was [sample child] ever treated or judged unfairly because of [his/her] race or ethnic group? )		Depression anxiety, and behavioral problems	· Adolescents who had been treated unfairly due to their race or ethnicity were significantly more likely than those who had not to have identified depression problems (OR = 3.75, 95% CI = 1.44–9.74, *p* < 0.01). · Adolescents who had experienced racial discrimination were significantly more likely to have anxiety problems, regardless of race, than adolescents who had not experienced racial discrimination (OR = 3.13, 95% CI = 1.58–6.19, *p* = 0.001). ·Adolescents who had experienced racial discrimination were more likely than adolescents who had not been to have identified behavior problems, regardless of race (OR = 2.94, 95% CI = 1.30–6.66, *p* < 0.01). Racial discrimination and ethnicity, the three controlled for variables of sex, health insurance, and poverty level were significantly associated with the likelihood of having identified behavior problems (*p* < 0.001).
Anderson et al., 2020	United States	Adolescents younger than 18 years *N* = 95,677	Cross sectional study	2011–2012 National Survey of Children’s Health (NSCH)	Parent or guardian	·A single item of experienced racial discrimination		Perception of health	· The proportion of adolescents with excellent health was 5.4% (95% CI = 3.6–7.2) lower with exposure to racial discrimination. Black and Hispanic adolescents showed significant associations between racial discrimination and general health in lower-income groups, while White adolescents showed a significant association in higher-income groups. ·In the structural equation model, racial discrimination, had significant indirect associations with general health through anxiety (effect= -0.08, 95% CI = 0.12–0.04) and depression (effect = -0.07, 95% CI = 0.11–0.03).
Cheon et al., 2020	United States	Ethnic/racial minority adolescents of Asian (41%), African American (22%), and Latinx (37%)/ (13–17 years) *N* = 350	Longitudinal study	Sampled from five ethnically/racially diverse New York City public high schools	Adolescent (self-report)	·Racial Ethnic Discrimination Index (e.g., I was treated unfairly because of my race/ethnicity over the past 6 months.)		Ethnic/racial identity	· The three profiles differed in the levels of prior discrimination experience (F (2, 335) = 3.57, *p* < 0.05). The first profile, which is “weakly identified”, was characterized by, low levels of Ethnic/racial identity (ERI) and American identity (AI). The second profile, which is “high ERI moderate AI”, by high levels of ERI, relatively moderate levels of AI. The third profile, which is ““moderate ERI and AI.” The “weakly identified” group scored the highest on prior discrimination experiences (M _weakly identified_ = 0.86, SD _weakly identified_ = 1.22; M _high ERI moderate AI_ = 0.41, SD _high ERI moderate AI_ = 0.70; M _moderate ERI and AI_ = 0.39, SD _moderate ERI and AI_ = 0.82).

^†^ OR = odds ratio

^‡^ CI = confidence interval

### Influences of discrimination on health and health behavior

Most studies measured discrimination using the widely implemented Everyday Discrimination Scale (EDS; *n* = 4, 36.4%) developed by Williams et al. [27], followed by the Discrimination Questionnaire (*n* = 2, 18.2%) developed by Stevens et al. [28]. One study each used the Racial Ethnic Discrimination Index (*n* = 1, 9.1%) and Adolescent Discrimination Distress Index (*n* = 1, 9.1%). Finally, three studies (27.3%) used single items to assess experiences of unfair treatment and discrimination. In terms of the type of discrimination, there have been many studies that measure whether people have been discriminated against because of his or her skin, color, origin, or religion; whether they have been treated less fairly than others in schools, stores, etc., whether they have been criticized by a particular ethnic group (Table 2).

In categorizing the health-related dependent variables, six studies (54.5%) measured mental health as an outcome affected by discrimination (e.g., psychiatric symptoms, the prevalence of psychotic experiences, depression, and anxiety). Overall health was measured as an outcome in two studies (18.2%), followed by substance use in two studies (18.2%), obesity (9.1%), behavioral problems (e.g., oppositional defiant disorder and conduct disorder) (9.1%), and ethic/racial identity in each study (9.1%).

All six studies that analyzed the effects of discrimination on mental health in adolescents reported that higher perceived discrimination was associated with worse mental health [29–34]. In one study among Moroccan-Dutch adolescents, personal discrimination (e.g., negative treatment based on their skin color, origin, and/or religion) was positively associated with psychiatric symptoms (odds ratio [OR], 2.69; 95% confidence interval [CI] [1.13, 6.41]) [29]. Looking at ethnic minorities in the Netherlands, El Bouhaddani et al. [31] similarly reported that personal discrimination was a predictor for psychotic experiences, including delusional and hallucinatory events (OR, 2.30; 95% CI: [1.22, 4.34]). Moreover, Adriaanse et al. [29] reported that psychiatric symptoms were associated with an increased odds of experiencing group discrimination, referring to whether adolescents felt that their ethnic groups were discriminated against in particular situations (e.g., stores, school, street locations, or during interactions with the police) (OR, 2.82; 95% CI: [1.57, 5.06]). Loyd et al. [32] investigated a sample of recently arrested ethnic minority adolescents and found that participants, who experienced episodes of interpersonal discrimination that were similar to group discrimination, reported higher internalizing symptoms (OR, 7.09; 95% CI: [3.95, 10.2]) and externalizing behaviors (OR, 7.87; 95% CI: [4.39, 11.3]). In sum, the investigated studies showed a clear association between group discrimination and mental health problems among ethnic minority adolescents.

Three studies (27.3%) reported that discrimination experience was a predictor for depression and anxiety among ethnic minority adolescents [30, 33, 34]. Even further, Pachter et al. [33] found that such experiences were significantly associated with higher levels of both 12-month major depression (OR, 1.21; 95% CI: [1.06, 1.39]) and 12-month anxiety (OR, 1.15; 95% CI: [1.00, 1.33]) in African-American and Afro-Caribbean adolescents. Similarly, Zapolski et al. [35] reported that discrimination had significant effects on symptoms of depression ($\:{\upbeta\:}=$ 0.22, *p* < 0.001) and anxiety ($\:{\upbeta\:}=$ 0.20, *p* < 0.001) in African-American adolescents.

Anderson et al. [35] identified that propensity score analysis shows that, unlike their white counterparts, experiences of discrimination are associated with poorer overall health outcomes for low-income Black and Hispanic adolescents group. As for long-term longitudinal research, Colen et al. [36] investigated a sample of upwardly mobile individuals from various racial/ethnic groups, finding that African-Americans and Latinos had significantly lower health outcomes than Whites despite being at the higher end of the socioeconomic spectrum. Moreover, upwardly mobile African-Americans and Latinos were surprisingly more likely to experience discrimination than their counterparts with lower socioeconomic status.

Some researchers also established that discrimination experiences were risk factors for substance use among ethnic minority adolescents [34, 37]. In Black Caribbean adolescents, Assari et al. [37] reported that higher discrimination was associated with higher substance use (OR, 1.15; 95% CI: [1.02, 1.29]), although there was no association between the level of discrimination exposure and smoking. Zapolski et al. [34] similarly reported that discrimination was a predictor of substance use ($\:{\upbeta\:}=$ 0.15, *p* < 0.001). Another study found that African-American fathers and their sons engaged in emotional eating in response to discrimination, which may degrade overall quality of life due to the risk of obesity [38]. Finally, Weeks and Sullivan [39] found that adolescents who were treated unfairly due to their ethnicity had a significant tendency for increased behavioral problems, regardless of the specific ethnicity (OR, 2.94; 95% CI: [1.30, 6.66]). Cheon et al. [30] conducted a latent profile analysis showing that ethnic/racial minority adolescents with prior discrimination experiences were more likely to have low levels of ethnic/racial identity and U.S. American identity (F = 3.57, *p* < 0.05).

### Gender differences in health/health behavior discrimination

Of the 11 reviewed studies, six (54.5%) investigated gender differences in health and/or health behaviors in adolescents based on discrimination experiences. Of these, two (33.3%) found relationships between gender and health/health behaviors, while the remaining four (66.7%) did not find significant gender differences in regard to discrimination experiences [30, 31, 34, 35]. Assari et al. [37] identified that high levels of discrimination were associated with higher substance use in male Caribbean Black adolescents (OR, 1.32; 95% CI: [1.05, 1.67]), with significant interactions between discrimination and gender on smoking (OR, 1.23; 95% CI: [1.07, 1.41]). Loyd et al. [32] found a significant interactions between ethnic/racial discrimination (i.e., experiences of discrimination in institutional, educational, and peer setting) and gender on internalizing symptoms including depression and anxiety (OR, -5.12; 95% CI: [-9.88, -0.35]); the association between interpersonal discrimination and internalizing symptoms was stronger among arrested female adolescents, primarily African-American.

### Quality appraisal

The majority of reviewed studies were well-designed and made appropriate efforts to reduce the risk of bias. As for the greatest concerns, the measurement of exposure appeared to pose a high risk of bias in three studies, while the blinding of outcome assessments showed an unclear risk of bias in all studies. Supplementary material 1 lists the results of the quality appraisal.

## Discussion

This study conducted a literature review to clarify how discrimination affected the lives of ethnic minority adolescents, particularly in regard to health status and health behavior outcomes. Interestingly, most of the studies were conducted in the U.S. American context, with the main research populations consisting of African-American adolescents. Indeed, previous systematic reviews have returned similar findings, in that most relevant studies were focused on minority populations in the United States [40, 41]. This highlights the need to investigate a greater diversity of research settings, especially to include areas with growing minority populations. For example, in 2021, ethnic minority children and adolescents accounted for 29.1% of Australia’s children and adolescents, with Indian and Chinese on the rise [42]. Consistent results emerged depending on whether the respondent was the parent or the adolescent themselves. In studies where parents reported on whether their child was treated unfairly because of race or ethnic group, adolescents who experienced discrimination were found to exhibit higher levels of depression, anxiety, and behavioral problems compared to their non-discriminated peers [39]. Similarly, research where adolescents self-reported on being teased because of race/ethnicity or skin color [34] and the adolescents’ self-reports on the Everyday Discrimination Scale [33] both concluded that depressive symptoms/disorders and anxiety symptoms/disorders were significantly higher, thus aligning with the findings of the parent-reported study.

Of particular note, most of the investigated studies reported that adolescents with discrimination experiences tended to have worse mental health such as psychiatric problems, psychotic experiences, anxiety, and depression. This suggests that racial discrimination is a major social determinant of health [40, 43], described as “where people live and grow” [9, 44]. According to the conceptualization of the social determinants of mental health, discrimination can lead to poor choices and adverse health behaviors, thus leading to risk factors such as substance use, poor dietary habits, and stress. In turn, exposure to adverse social conditions increases the risk of poor mental health [43]. Further, negative health impacts stemming from discrimination result in health disparities. As the unequal distributions of opportunities, resources, and healthcare access are driven by social norms and public policies [43], public health professionals should establish and implement interventions designed to help ethnic minority adolescents who have experienced discrimination. Colen et al. [36] analyzed longitudinal data of adolescents, finding that despite African Americans and Hispanics being higher on the socioeconomic status, their health status were substantially poorer than those of Whites. This aligns with research from 1999 to 2018 showing that, regardless of income, the health status of Black people was consistently lower than that of White people [45]. The results affirm the notion that race or ethnicity exerts a more significant influence on health inequalities than socioeconomic status. As race and ethnicity are inherent traits, it underscores the necessity of policy-driven initiatives aimed at enhancing healthcare access for these communities to alleviate such disparities.

The perception of discrimination was also associated with substance use, and increased body mass index in selected studies, all of which are indicators of worsening health [46]. Moreover, some researchers found gender differences in substance use rates [37], which is consistent with previous findings that discrimination lead to increases in substance use for African American male adolescents [47]. Preventive intervention related to discrimination can be applied in school settings, since study of Brody et al. [47] have shown that school engagement has a mediating effect between discrimination and substance use. Looking back at gender, there is evidence that female adolescents who are exposed to discrimination tend to internalize symptoms such as depression rather than externalizing their symptoms through behaviors such as substance use [48], which points to the need for gender considerations in these interventions. Previous study found that the high emotional eating in African American youth (aged 18–27) with high frequency of the discrimination experience [49] is similar to the results of discrimination and relationship of emotional eating in this study.

We also identified a relationship between discrimination and low-level health perceptions. This is consistent with the results of a previous systematic review showing a significant and consistent relationship between racism and ill health [4]. As Colen et al. [36] found that Black adolescents were more vulnerable than Hispanic adolescents when recovering from discrimination experiences, interventions designed to alleviate racial disparity may require tailored approaches.

A comparison of studies on adolescent discrimination and health, conducted before [13] and after 2016 (as reported in this study), shows consistent trends. Most studies were conducted in the United States, followed by Europe. Studies prior to 2016 found strong links between discrimination and depression, internalizing symptoms, and externalizing behaviors. Similarly, six of 11 studies since 2016 reported that discrimination was associated with increased depression, anxiety, and, in severe cases, psychiatric symptoms and psychotic experiences, emphasizing its harmful effects on mental health. Prior to 2016, the association between discrimination and substance use was weak, and similarly, only two studies found a significant association after 2016. Gender differences in the impact of discrimination on socioemotional well-being were not significant before 2016, and only two post-2016 studies reported differences: one linked higher depression and anxiety to females, while the other connected higher substance use to male adolescents. These results highlight the need for further research on gender-specific health outcomes and meta-analytical reviews. Notably, studies since 2016 identified increased relations of oppositional defiant disorder, conduct disorder, and emotional eating among adolescents exposed to discrimination—findings not reported in earlier research, highlighting the evolving understanding of the broader health impacts of discrimination on adolescents.

Future studies should have public health professionals, proactively implement strategies to provide professional education; moreover, research should be conducted for reducing the negative health effects of structural, personally mediated, and internalized racism and improving the well-being of adolescents [50]. This is especially important for improved health and health care in minority groups [12].

### Implications for practice

This article will contribute to providing evidence-based practice for public health professionals, as advocacy may be critical for ethnic minority adolescents. Although discrimination was found to have the greatest effect on mental health in this study, it was also identified to have an impact on a variety of health behaviors including substance use and emotional eating. Therefore, it is necessary to develop and provide an integrative health prevention program for ethnic minority adolescents in the community setting. School health professionals in particular can play an important role in alleviating the experiences of discrimination among ethnic minority adolescents and preventing negative impact on health status and behaviors. Learning from the experience of COVID-19, where inaccurate information led to discriminatory attitudes toward certain ethnicities [18], school health professionals should communicate accurate information about the issue to mitigate racial and ethnic discrimination. Policymakers should increase public awareness about discrimination through educational programs and campaigns, thus preventing its widespread occurrence. Moreover, Gender differences in the effects of discrimination on health status and health behavior have been identified in only few studies and should be continued in future studies. It is expected to apply gender specific intervention considering these study findings that male adolescents who have experienced discrimination revealed have higher substance use than female adolescents, and that female adolescents who have experienced discrimination are higher internal symptoms than male adolescents.

### Limitations

All studies selected for this integrative review were observational studies, of which 8 studies (72.7%) were cross sectional studies, with limited research design. As we reviewed publications published within the last 5 years, ethnic backgrounds may have been limited, so caution should be taken in generalizing the results. In addition, all of the Blinding of Outcome Assessment in the Quality Appraisal have been assessed as unclear risk of bias, meaning that it has been studied only in cross-sectional studies among quantitative research in the last five years. The results of this study have strength that provides evidence in the needs to perform experimental and longitudinal studies about ethnic minority adolescents’ discrimination on health and health behavior. The possibility that studies other than four databases and written in English were excluded is the limitation of this study.

## Conclusion

This integrative review provides insights into the discrimination experiences of ethnic minority adolescents, with particular implications for mental health, overall health, substance use, behavioral problems, and important gender differences. In turn, this underscores the need for public health professionals to recognize the health vulnerabilities of adolescents due to their racial and ethnic identity and to be concerned with protecting them from factors associated with experiences of discrimination. The findings emphasize that discrimination during adolescence has a particularly strong impact on the health of ethnic minorities with lower socioeconomic status. The fact that economic success does not moderate the impact on health underscores the importance of prioritizing research on discrimination during this critical developmental period. Because efforts to identify gender disparities in the impact of discrimination on health and risky behaviors have yielded limited results, these findings are inconclusive and further research is needed to examine gender-specific effects.

## Electronic supplementary material

Below is the link to the electronic supplementary material.

Supplementary Material 1

## Data Availability

Not applicable.
