# Supplementary material for: The recent trends in discrimination and health among ethnic minority adolescents: an integrative review
Source: BMC Public Health. 2025 Mar 4;25:861. doi: 10.1186/s12889-025-21729-0 (PMC11877704; doi:10.1186/s12889-025-21729-0)
Supplement: Supplementary file 1 — Supplementary Material 1 [file 12889_2025_21729_MOESM1_ESM.docx]

**Supplementary Material 1**. Results of Quality Appraisal

| **Criteria** | **Adriaanse et al. 2016** | **Assari**  **et al. 2018** | **Colen**  **et al. 2018** | **Pachter**  **et al. 2018** | **El Bouhaddani et al. 2019** | **Zapolski et al.**  **2018** | **Coleman et al. 2019** | **Loyd**  **et al. 2019** | **Weeks**  **et al. 2019** | **Anderson et al. 2020** | **Cheon**  **et al.**  **2020** |
| --- | --- | --- | --- | --- | --- | --- | --- | --- | --- | --- | --- |
| Type of Study | Cross Sectional | Cross Sectional | Longitudinal | Cross Sectional | Longitudinal | Cross Sectional | Cross Sectional | Cross Sectional | Cross Sectional | Cross Sectional | Longitudinal |
| Selection of Participants | **+** | **+** | **+** | **+** | **+** | **+** | **-** | **+** | **+** | **+** | **+** |
| Confounding Variables | **?** | **+** | **+** | **+** | **+** | **+** | **+** | **+** | **+** | **+** | **-** |
| Measurement of Exposure | **+** | **+** | **+** | **+** | **-** | **+** | **+** | **+** | **-** | **-** | **+** |
| Blinding of Outcome Assessments | **?** | **?** | **?** | **?** | **?** | **?** | **?** | **?** | **?** | **?** | **?** |
| Incomplete Outcome Data | **?** | **+** | **+** | **+** | **+** | **+** | **+** | **+** | **+** | **+** | **+** |
| Selective Outcome Reporting | **+** | **+** | **+** | **+** | **+** | **+** | **+** | **+** | **+** | **+** | **+** |

|  | **Key** |
| --- | --- |
| **+** | Low Risk of Bias |
| **?** | Unclear Risk of Bias |
| **-** | High Risk of Bias |
